# Shared medical appointments and patient-centered experience: a mixed-methods systematic review

**DOI:** 10.1186/s12875-019-0972-1

**Published:** 2019-07-08

**Authors:** Kim H. Wadsworth, Trevor G. Archibald, Allison E. Payne, Anita K. Cleary, Byron L. Haney, Adam S. Hoverman

**Affiliations:** 1 Pacific Northwest University of Health Sciences, College of Osteopathic Medicine, Yakima, WA USA; 2 Family Health Care of Ellensburg, Ellensburg, WA USA; 3 Multnomah County Health Department, Oregon Health and Science University–Portland State University School of Public Health, Portland, OR USA

**Keywords:** Shared medical appointment, Group visit, Cooperative health care clinic, Group prenatal care, Patient satisfaction, Patient experience, Health services, Primary care, Primary health care, Coproduction

## Abstract

**Background:**

Shared medical appointments (SMAs), or group visits, are a healthcare delivery method with the potential to improve chronic disease management and preventive care. In this review, we sought to better understand opportunities, barriers, and limitations to SMAs based on patient experience in the primary care context.

**Methods:**

An experienced biomedical librarian conducted literature searches of PubMed, Cochrane Library, PsycINFO, CINAHL, Web of Science, ClinicalTrials.gov, and SSRN for peer-reviewed publications published 1997 or after. We searched grey literature, nonempirical reports, social science publications, and citations from published systematic reviews. The search yielded 1359 papers, including qualitative, quantitative, and mixed method studies. Categorization of the extracted data informed a thematic synthesis. We did not perform a formal meta-analysis.

**Results:**

Screening and quality assessment yielded 13 quantitative controlled trials, 11 qualitative papers, and two mixed methods studies that met inclusion criteria. We identified three consistent models of care: cooperative health care clinic (five articles), shared medical appointment / group visit (10 articles) and group prenatal care / CenteringPregnancy® (11 articles).

**Conclusions:**

SMAs in a variety of formats are increasingly employed in primary care settings, with no singular gold standard. Accepting and implementing this nontraditional approach by both patients and clinicians can yield measurable improvements in patient trust, patient perception of quality of care and quality of life, and relevant biophysical measurements of clinical parameters. Further refinement of this healthcare delivery model will be best driven by standardizing measures of patient satisfaction and clinical outcomes.

**Electronic supplementary material:**

The online version of this article (10.1186/s12875-019-0972-1) contains supplementary material, which is available to authorized users.

## Background

Shared medical appointments (SMAs), or group visits, are a healthcare delivery innovation arising from the changing demands of patient-centered medical home (PCMH) settings and the primary care context. The model emphasizes prompt access and improved service, increased doctor-patient contact time, greater patient education, enhanced prevention and disease self-management, closer attention to routine health maintenance and performance measures, and the central role of patient and clinician experience within the Triple Aim: enhancing patient experience, improving population health, and reducing costs [1–3]. More recently, Bodenheimer and Sinsky recommended that “the Triple Aim be expanded to a Quadruple Aim, adding the goal of improving the work life of health care providers, including clinicians and staff [4].”

We chose SMA as the overarching term to encompass shared visit, group appointment, group medical appointment, group visit (GV), group medical clinic, shared in-group medical appointment, group prenatal care (GPNC) and group-based antenatal care. SMAs prioritize the delivery of care within interprofessional environments utilizing peer-to-peer interactions [5]. Multiple standardized SMA delivery models have been established, from the drop-in group medical appointment, cooperative health care clinic (CHCC) and physicals shared medical appointment, to CenteringPregnancy® (CP) and parenting visits [3, 6]. These visits frequently emphasize the “coproduction” roles of patients as experts in their own circumstances and health professionals as facilitators rather than fixers, thus fostering a shared experience of illness and health to better inform, empower, and support [2].

SMAs have garnered a body of evidence in chronic disease management and preventive care. The various interpretations of the group clinical model have been applied to a wide array of settings and a myriad of health promotion and disease-focused visits, including patients with diabetes, hypertension, congestive heart failure, chronic lung disease, asthma, arthritis, stroke, kidney disease, cancer, hearing impairment, and prenatal care, among other conditions [7–15].

Several systematic reviews summarize the effects of SMAs on healthcare delivery, economic factors, and biophysical outcomes. Health systems have begun to embrace the need for this transformative approach in achieving patient goals [2, 16–18]. In an era recognizing the role of patient-centeredness in improving healthcare quality, numerous authors have highlighted the need for a review that addresses the impacts of SMAs on patient experience of care [3, 7, 16, 17, 19]. This review aims to meet this need by examining the patient experience from the published literature alongside an assessment of SMAs to improve biophysical outcomes in the adult primary care setting.

Analyzing the existing body of evidence for shared medical appointments, we sought to understand the opportunities, barriers, and limitations to SMAs based on self-reported patient experience, a notable component of the Triple Aim [2]. Specifically, our goal was to highlight effective approaches for patients participating in SMAs and determinants of effectiveness.

## Methods

An experienced biomedical librarian conducted preplanned literature searches of PubMed, Cochrane Library, PsycINFO, Cumulative Index of Nursing and Allied Health Literature (CINAHL), Web of Science, ClinicalTrials.gov, and Social Science Research Network (SSRN) for peer-reviewed publications, using controlled vocabulary, keywords, and text words (see Additional file 1 for search strategy details). The search was limited to publications from 1997 or after. We also searched grey literature, non-empirical reports, social science publications, and citations from published systematic reviews. The search yielded 1359 papers, including qualitative, quantitative, and mixed-methods studies. Case studies, pilot/feasibility studies, protocols, opinions, or advocacy articles were excluded. Eligibility criteria and methods of analysis were specified a priori.

Two researchers independently reviewed citation titles, abstracts, and full-text articles to determine eligibility as well as extracted the data and performed quality and risk of bias assessment on included articles, as detailed below. Before general use, we pilot-tested the abstraction form templates on a sample of included articles and then revised accordingly to ensure that all relevant data elements were captured. Disagreements were resolved by consensus of the two reviewers or by obtaining a third investigator’s opinion when consensus could not be reached.

Studies were required to meet five process (p) and outcome (o) criteria: clinical intervention (o), clinician-led visit (p), patient experience of care (o), primary care (p), and availability of individual clinical consultation (p), as detailed below. Studies were excluded if any participants were < 18 years of age. To limit potential bias, we excluded studies involving addiction medicine, substance dependence / rehabilitation treatment, inpatient settings (both short and long term) or chronic care clinics that implemented multiple interventions, and SMAs requiring management by a specialist.

We deemed SMAs to be clinician led if led by an independent licensed prescriber or clinician. This included medical doctors (MDs), doctors of osteopathy (DOs), advanced registered nurse practitioners (ARNPs), certified nurse midwives (CNMs), and in some regions, nurse practitioners (NPs). We verified prescriptive authority and care responsibility by consulting organizational websites from the countries in which our identified studies were conducted [20–22].

Our review emphasized biophysical metrics of adult patients in primary care environments. The study team included articles focused on SMAs that implemented a clinical intervention, such as vital sign measurements, lab checks (e.g., hemoglobin A1c, lipid panels), medication adjustments, or physical exams. We excluded studies if the intervention was limited to patient education, facilitation, peer-facilitated support groups, or group talk therapy.

We tracked confounders within targeted studies, such as participant inclusion/exclusion criteria, local barriers to implementation, reimbursement framework, types of SMA interventions, and patient characteristics including language, culture, and socioeconomic status.

In our consideration of quantitative research, we included only those studies with a comparative control group. Studies with quantitative primary outcomes were evaluated using the modified Jadad score, which assesses the overall quality of the individual studies, including risk of bias, and has shown high inter-rater reliability [23–26].

To evaluate qualitative studies, our team used the “Trustworthiness of Qualitative Inquiry” framework to assess credibility, transferability, dependability, and objectivity [27].

Inter-rater reliability was assessed during the data extraction phase via two-way mixed measures intraclass correlation (ICC) value for average agreement presented [28].

In consideration of ENTREQ and PRISMA frameworks for this mixed-methods systematic review, categorization of the extracted data informed a thematic synthesis [29–32]. We did not perform a formal meta-analysis.

## Results

**Fig. 1:**
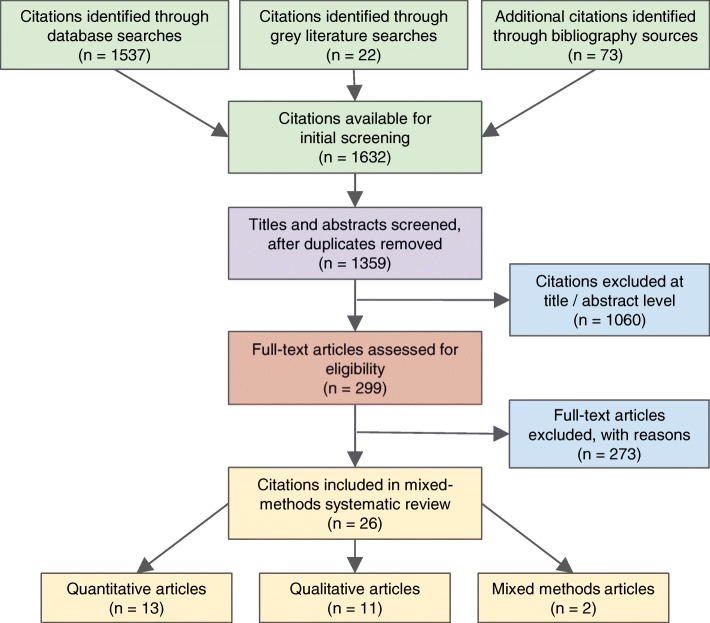
The PRISMA flowchart for all included studies

Thirteen quantitative controlled trials, 11 qualitative papers, and two mixed methods studies met inclusion criteria. Three models were identified: CHCC (five articles), SMA / GV (10 articles) and GPNC / CP (11 articles). Figure 1 shows the Preferred Reporting Items for Systematic Reviews and Meta-Analyses (PRISMA) flowchart for all included studies [32].

### Summary of included studies

SMA / GV is the most frequently mentioned model in quantitative studies whereas the GPNC / CP model is the most common in qualitative studies in this review. The CHCC model is the least represented in this review (Table 1).

**Table 1:** List of 26 included articles in the primary care setting, categorized by model of group clinic and study type

Model:	CHCC	SMA / GV	GPNC / CP
Quantitative (13 articles)			
Beck, 1997	X		
Clancy, 2007	X		
Jafari F, 2010			X
Junling, 2015	X		
Kennedy, 2011			X
Naik, 2011		X	
Scott, 2004	X		
Tandon, 2013			X
Trento, 2001		X	
Trento, 2002		X	
Trento, 2004		X	
Trento, 2005		X	
Trento, 2010		X	
Qualitative (11 articles)			
Andersson, 2012			X
Andersson, 2013			X
Capello, 2008		X	
Clancy, 2003	X		
Herrman, 2012			X
Kennedy, 2009			X
McDonald, 2014			X
McNeil, 2012			X
Novick, 2011			X
Raballo, 2012		X	
Wong, 2015		X	
Mixed-methods (2 articles)			
Heberlein, 2016			X
Krzywkowski-Mohn,2008		X	
Total no. of articles (26)	5	10	11

Abbreviations: *CHCC* Cooperative health care clinic, *CP* CenteringPregnancy®, *GPNC* Group prenatal care, *GV* Group visit, *SMA* Shared medical appointment

Table 2 breaks down the included articles into locale, healthcare system, reimbursement model, study design, single site or multiple sites, and study duration.

**Table 2:** Characteristics of included studies in the primary care setting

Study characteristics	*N* studies (participants)			
	Diabetes	HTN	MCC	Pregnancy
No. of studies, by medical condition	10 (1881)	2 (1262)	3 (645)	11 (2010)
Country				
United States	4 (426)	1 (58)	2 (616)	6 (926)
Canada	0	0	1 (29)	2 (21)
Europe (Italy, Sweden)	6 (1455)	0	0	2 (435)
Middle East (Iran)	0	0	0	1 (628)
Asia (China)	0	1 (1204)	0	0
Healthcare system				
Govt (VA, FQHC, NHS, PHD)	3 (362)	2 (1262)	1 (29)	8 (1908)
Private (HMO, MCO)	1 (120)	0	2 (616)	3 (102)
University-affiliated clinic	6 (1399)	0	0	0
Healthcare payment model				
Public (Medicaid, Medicare, govt funded)	8 (1575)	2 (1262)	3 (645)	8 (1908)
Private (fee-for-service, managed care)	0	0	0	3 (102)
Uninsured /underinsured	2 (306)	0	0	0
Study design				
Randomized controlled trial	9 (1848)	1 (1204)	2 (616)	4 (1591)
Non-randomized controlled trial	0	0	0	1 (268)
Observational / interviews / focus groups	1 (33)	1 (58)	1 (29)	5 (122)
Mixed methods	0	0	0	1 (29)
Sites				
Single	9 (1066)	1 (58)	1 (321)	4 (84)
Multisite	1 (815)	1 (1204)	2 (324)	7 (1926)
Study duration				
< 6 months	1 (87)	1 (1204)	0	0
6 months	1 (120)	1 (58)	0	0
7 to 11 months	0	0	0	11 (2010)
12 to 18 months	2(219)	0	2 (350)	0
24 months	3 (1169)	0	1 (295)	0
> 2 years	3 (286)	0	0	0

Abbreviations: *FQHC* Federally qualified health center, *HMO* Health maintenance organization, *HTN* Hypertension, *MCC* Multiple chronic conditions, *MCO* Managed care organization, *NHS* National health service, *PHD* Public health district, *VA* Veterans Administration

Table 3 provides details of the typical configuration of the three models included in this review: CHCC, SMA / GV, and GPNC / CP. Generally, CHCC has a larger group size compared to SMA / GV and GPNC / CP. Physician-led intervention teams were cited in most SMA / GV studies, whereas certified nurse midwives were most often cited as leaders of the GPNC / CP visits.

**Table 3:** Typical configuration of group models, as represented by included studies in the primary care setting

Model (no. of articles)	Duration of each group session	Duration of individual consultation	Group size	Clinical intervention	Nonclinical components	Intervention team	
						Disciplines (no. of articles)	Size
CHCC (5)	90–120 min	5–10 min each at end of group session	6–20	Vital signs Lab results review and medical records update Medication management Preventive measures Scheduling Medical-related paperwork requested by pts Brief 1:1 visits with physician, as necessary	Socialization Health education Group cohesion	PCP (5) Nurse, RN or diabetes nurse educator (5) Clinical pharmacist (2) PT, OT (2) Dietitian (2) Community health worker (1)	2–5
SMA / GV(10)	60–90 min	Optional 10 mins each or 24 mins total allotted at end of group session	5–15	Vital signs Lab results review and medical records update Routine lab test orders 1:1 indiv consultation with physician, as necessary Health risk assessment Medication management Referrals, coordination of public health services	Orientation and socialization Interactive health education Group cohesion Self-monitoring Group discussion Medication compliance	1–2 physicians (9) Nurse, NP, RN (2) Diabetes educator/ RD (4) Clin psychologist, psychopedagogist (3) 1–2 postgraduate med students (1) Others (2)	2–7
GPNC / CP^a^(11)	90–120 min	10 mins each at beginning of group session	8–12	Vital signs Physical exam Routine prenatal screening and labs Routine ultrasound Flu vaccine (seasonal) Postpartum visit Individual assessments prior to prenatal care within group setting	Group discussion, self-care, skills-building Active tracking of pregnancy changes (done by pts) Tour of birth unit, labor and delivery nurse Pediatric care resources Postpartum reunion	1–2 CNMs (8) NP (3) Medical asst (3) Physician (2) Health / perinatal educator (1) Others (1)	2 + others invited

Abbreviations: *CHCC* Cooperative health care clinic, *CNM* Certified nurse midwife, *CP* CenteringPregnancy®, *GPNC* Group prenatal care, *GV* Group visit, *NP* Nurse practitioner, *OT* Occupational therapist, *PCP* Primary care physician, *PT* Physical therapist, *RD* Registered dietitian, *RN* Registered nurse, *SMA* Shared medical appointment

^a^Wk 5–10: First visit w/ nurse. Wk 10–12: First visit with clinician. Wk 12–16: Start CP program

Per inclusion criteria, all 26 articles reported patient satisfaction and experience (Table 4). Only one article reported outcomes for all four aims [8].

**Table 4:** Quadruple aim reported in included studies

Model (no. of articles)	No. of articles			
	Patient experience	Population health	Cost	Clinician experience
CHCC (5)	5	2	2	3
SMA / GV (10)	10	1	1	3
GPNC / CP (11)	11	3	0	1

Abbreviations: *CHCC* Cooperative health care clinic, *CP* CenteringPregnancy®, *GPNC* Group prenatal care, *GV* Group visit, *SMA* Shared medical appointment

### Patient experience and satisfaction

Methodologies for tracking patient experience and satisfaction were grouped by data collection method into the following five categories: One-on-One Interviews (via telephone or in person), Focus Group Style Interviews, Self-Efficacy / Participation / Satisfaction Questionnaires, Diabetes-Related Quality of Life (DQoL) Related Scales; and Primary Care Assessment Tool / Trust in Provider Outcomes (Table 5).

**Table 5:** Methods used to collect patient experience data

Method	No. of articles
1:1 phone or in-person interviews^a^	10
Focus group style interviews^a^	3
Self-efficacy / participation / satisfaction questionnaires	6
Diabetes-related quality of life scales (DQoL)	6
Primary care assessment tool & trust in clinician outcomes	2
Total:	27

^a^Andersson 2012 is double coded as it included both 1:1 and group interviews

When comparing the results of the patient experience / satisfaction data in these 26 articles, the following six major themes emerged (also see Additional file 2 for more details).

### Patient-clinician dynamic

Overall, data on the patient-clinician dynamic that emerged during SMAs were positive. SMAs saw quantitative advantages over individual visits in domains ranging from improved communication to overall satisfaction with the visit [7, 15, 33]. In SMA environments, more time was allotted to discuss healthcare issues with the clinician compared to traditional individual visits, and physicians were perceived as less hurried [7, 14]. One study indicated that SMA experiences resulted in markedly enhanced trust in one’s primary care physician [33].

Qualitative feedback similarly supported the patient-clinician dynamic as a notable aspect of SMAs. Interviews with CP patients indicated that extra time with clinicians helped them to develop strong, supportive, and positive relationships with their healthcare clinicians, and reduced anxiety about potentially not being familiar with the practitioner who would oversee their obstetric deliveries [9–11, 34].

Feedback from patients indicated that room for further improvement of the patient-clinician dynamic in SMAs lies in the avoidance of a paternalistic, didactic style of communication from the clinician leader [12]. Patients appreciated being empowered by their clinicians and preferred a more encouraging and empowering communication style within their groups.

### Overall quality of care

Multiple studies demonstrated that patients participating in SMAs were significantly more satisfied with their care than those in individual models of care [7, 13–15]. When compared to patients receiving traditional individual care, those participating in SMAs were more likely to describe their overall quality of care as excellent, to feel that their care was meeting all their needs, and to feel that their care was well coordinated [8, 35]. No studies showed significant decreases in patient perceptions of quality of care in SMAs.

Overall quality of care was not a direct theme extracted from qualitative investigations of SMAs. However, interviews from Herrman’s research on the CP program revealed that “multiparous women frequently commented that [SMAs were] far superior to their previous experiences” [11].

### Quality of life

Trento’s research thoroughly addressed the theme of quality of life, using a modified version of the Diabetes Quality of Life Measure (DQoL) questionnaire consisting of 39 questions ranked along a 5-point Likert scale. This assessment scale was used across all five of Trento’s articles, and demonstrated consistent results over 10 years of varied research on SMAs for patients with Diabetes Mellitus, Type 2 (T2DM). In all five of Trento’s studies discussed in this paper, DQoL scores significantly improved among group participants while worsening or remaining the same in control subjects [36–40].

### Sense of community

Patients in multiple studies reported that the feeling that they were not alone in their experience was central to the positive impact of SMAs and persisted whether the subject of the SMA was pregnancy, navigation of the VA system, or hypertension [6, 10, 12, 33, 41–44]. Creation of community via SMAs supported patients’ emotional health by providing validation and stemming the isolation often experienced when managing chronic conditions. This sense of community was viewed as a benefit, though one study referenced a member who reported that at times she avoided discussion of “disturbing topics for fear that it would negatively impact her cohort” [34].

### Patient empowerment ***/*** role in healthcare

This body of research suggests that a strength of SMAs over usual care is the ability to engage and empower patients as active participants in their own healthcare. This empowerment bore out in both qualitative and quantitative research participants. Quantitatively, patients reported that they were more able to participate in their care and had significant improvements on scales of Coping Skills and Health Distress as compared to their counterparts [13, 14, 43]. In the realm of qualitative analyses, it was described that patients felt they were better able to interpret their medical data, thus making them more likely to discuss their issues with their clinicians [42]. Within the CP model, patients reported feeling “reassured, prepared, less anxious, and confident,” and they felt that the group sessions made them more proactive with respect to their own health [9]. Raballo’s research also indicated that after experiencing SMAs, patients were significantly more likely to describe an internal locus of control for their health than those followed by usual care [45].

### Access / efficiency

Several articles also establish benefits of SMAs with respect to access and efficiency. Quantitatively, participants reported that appointments were easily scheduled “as soon as [they liked]” and were more likely to report that visit waiting time was acceptable [8, 14]. Qualitatively, patients described experiencing “more comprehensive services,” smoother communication between clinicians, decreased waiting times, increased opportunities for learning throughout their visits, and improved administrative support [41, 42, 46].

### Biophysical outcomes

Less than half of the included articles reported biophysical outcomes by health condition—either diabetes mellitus (DM) or hypertension (HTN)—as summarized in Table 6 [36–40, 42, 43, 45, 47, 48]. These studies claimed significant and non-significant improvements in biophysical metrics; however, heterogeneity of study populations, methods and outcomes did not allow data across studies to be combined and analyzed.

**Table 6:** Overview of biophysical data from available studies, categorized by health condition (no. of articles = 10)

First author, year	HbA1c	FBG	Lipids	BP	BMI	Body wt	CV risk	DM Rx dosage	Kidney	Eye	Foot	Physical activity
Diabetes												
Trento, 2001	X		X HDL, TG		X							
Trento, 2002	X		X HDL	X	X	X	X	X		X retinopathy		
Trento, 2004	X		X HDL, TG		X				X Cr			
Trento, 2005	X	X	X TC, HDL, TG		X	X		X insulin	X ACR		X foot ulcers	
Trento, 2010	X	X	X TC, LDL, HDL, TG	X	X				X Cr			
Naik, 2011	X			X SBP	X							
Raballo, 2012	X	X	X TC, HDL, TG		X							
Krzywkowski-Mohn, 2008	X		X LDL	X						X retinal exam	X foot exam	
Hypertension												
Junling, 2015				X	X							X
Capello, 2008				X								

Abbreviations: *ACR* Albumin/Creatinine ratio, *BMI* Body mass index, *BP* Blood pressure, *Cr* Creatinine, *CV* Cardiovascular, *DM* Diabetes mellitus, *FBG* Fasting blood glucose, *HbA1c* Glycated hemoglobin, *HDL* High-density lipoprotein, *LDL* Low-density lipoprotein, *Rx* Prescription, *SBP* Systolic blood pressure, *TC* Total cholesterol, *TG* Triglycerides

This data subset was categorized into quantitative (seven articles), qualitative (two articles), and mixed methods (one article) studies to include additional details (Table 7). Eight articles had a control comparator of usual care while two articles (one qualitative study and one mixed methods study) only compared pre- and post-group intervention. Only one article utilized the CHCC model while the remaining nine articles were SMAs / GVs. From the ten studies included in this subset, the reported biophysical profile data varied, keeping with previous systematic reviews on SMAs by Booth et al. and Edelman et al. [17, 18].

**Table 7:** Biophysical data from available studies, categorized by research type (no. of articles = 10)

First author, year	Model	Health cond(s)	Sample size (n)	Biophysical measures	Reported findings (with *p*-values)
Quantitative					
Junling, 2015	CHCC	HTN	600 group, 604 control	● BP	SBP decreased significantly in both group (p < 0.001) and control (*p* = 0.001) from baseline to follow-up, although decreases in group > control.
				● BMI	
					DBP decreased significantly in group (p = 0.001) but did not decrease significantly in control.
				● Physical activity	
					BMI did not change in both.
					Increases in physical activity in group (p < 0.001) more remarkable than in control.
Trento, 2001	SMA / GV	T2DM	56 group, 56 control	● HbA1c	HbA1c stable in group, worsened in control (*p* < 0.002).
				● BMI	Tendency toward lower BMI in group (*p* = 0.06).
				● HDL	HDL cholesterol initially similar in both but later lower in group only (*p* < 0.05).
				● Fasting TG	Trend toward lower TG in group (*p* = 0.053).
Trento, 2002	SMA / GV	T2DM	56 group, 56 control	● Dosage of anti-hyperglycemic agents ● Body wt, BP and CV risk ● Metabolic control: - HbA1c - BMI - HDL - Retinopathy	Dosage of hypoglycemic agents decreased (p < 0.001) among group compared to control. Body wt (p < 0.001) and BMI (*p* < 0.001) decreased in group but not in control. Similar reductions in BP and CV risk in group vs control, but diff significant only for DBP (p < 0.001). Significant decrease in HbA1c (p < 0.001) in group. HDL increased (p < 0.001) in group but not in control. Retinopathy progressed less in group (*p* = 0.009).
Trento, 2004	SMA / GV	T2DM (NIDDM)	56 group, 56 control	● HbA1c ● BMI ● HDL, TG ● Cr	HbA1c remained stable in group but progressively increased among control (*p* < 0.001). BMI, HDL, TG and Cr improved over 5 yrs. in group, but not significantly different from control.
Trento, 2005	SMA / GV	T2DM	31 group, 31 control	● HbA1c	HbA1c decreased in both, though not significantly.
				● Lipids (TC, HDL, TG)	TC decreased in controls (p < 0.05), while HDL increased in group (*p* = 0.027).
				● Body wt, BMI	No significant modifications in other clinical variables monitored (body wt, BMI, FBG, insulin dosage, TG, ACR, foot ulcers).
				● FBG	
				● Insulin dosage	
				● ACR	
				● Foot ulcers	
Trento, 2010	SMA / GV	T2DM (NIDDM)	421 group, 394 control	● FBG ● HbA1c	FBG, HbA1c, TC, TG, LDL cholesterol, SBP, DBP, and BMI decreased in group from baseline to year 4 compared to control (p < 0.001, for all measures). HDL increased in group (p < 0.001).
				● TC, LDL, HDL, TG	Cr did not change significantly in group. BMI, HbA1c, TG, and Cr increased in control, whereas total, HDL, and LDL cholesterol and SBP did not change and DBP decreased.
				● BP	
				● BMI	
				● Cr	
Naik, 2011	SMA / GV	T2DM	45 group, 42 control	● HbA1c ● SBP ● BMI	Significantly greater improvements in HbA1c immediately following active Intervention and persisted at 1-year follow-up (p = 0.05). SBP and BMI were only reported at baseline, but not significantly different between both.
*Qualitative*					
Capello, 2008	SMA / GV	HTN	58 group (no control)	● BP	Significant effects on SBP and DBP (*p* < 0.01).
Raballo, 2012	SMA / GV	T1DM, T2DM	121 group, 121 control	● HbA1c ● Lipids (TC, HDL, TG) ● FBG ● BMI	HbA1c lower in T1DM group than in control (p = 0.001) and not significantly so in T2DM (NS). Lower HDL in T1DM control (*p* = 0.002), but no other significant differences among both.
*Mixed Methods*					
Krzywkowski-Mohn, 2008	SMA / GV	T2DM	33 group (no control)	Diabetic clinical indicators:	
				● HbA1c	Lower HbA1c after group intervention (p < 0.05).
				● LDL	Lower LDL after 18 mos (p < 0.05).
				● BP	No significant diff. in SBP or DBP after 18 mos.
				● Retinal exam	Increase in diabetic eye exams.
				● Foot exam	No diff in diabetic foot exams (96.9% pre + post).

Abbreviations: *ACR* Albumin/Creatinine ratio, *BMI* Body mass index, *BP* Blood pressure, *Cr* Creatinine, *CV* Cardiovascular, *DBP* Diastolic blood pressure, *FBG* Fasting blood glucose, *HbA1c* Glycated hemoglobin, *HDL* High density lipoprotein, *HTN* Hypertension, *LDL* Low density lipoprotein, *NIDDM* Non-insulin dependent diabetes mellitus, *SBP* Systolic blood pressure, *T1DM* Diabetes mellitus, type 1, *T2DM* Diabetes mellitus, type 2, *TC* Total cholesterol, *TG* Triglycerides

### Barriers to implementation

Few studies addressed barriers, as shown in Additional file 3. Prior reviews by Edelman et al., Booth et al., and Jones et al. cite several barriers to implementation of SMAs overall, including patient participation and attendance, group dynamic incompatibilities, cost-benefit concerns, and staff/facilities inadequacies [16, 17, 49].

Prior studies cited poor attendance at SMAs [7, 13, 33]. In tracking attendance and patient-centered outcomes through different group visit formats, durations and patient populations, a great variation of attendance rates was found, as shown in Additional file 4.

### Inter-rater reliability

As shown in Additional file 5, the ICC(2,k) inter-rater reliability values are 0.956 for Jadad-modified score of quantitative studies, 0.923 for trustworthiness score of qualitative studies, and indeterminable for mixed method studies due to sample size of *n* = 2 studies. Values greater than 0.90 indicate excellent reliability [28].

## Discussion

This review limited SMA models to three general categories: cooperative health care clinic, shared medical appointment / group visit, and group prenatal care / CenteringPregnancy®. To meet the focus on group clinical intervention, we considered visits that included the following clinical components: review of labs, medication management, physical examination, or other medical interventions. From a strength of evidence perspective, 16 of the studies reflected a randomized controlled design and one non-randomized controlled design. The remaining nine studies were cohort and case study designs, with a median study duration of 12 months.

As SMAs are generalizable to primary care environments, we prioritized reviews that included Internal Medicine, Obstetrics/Gynecology, Family Medicine, and Psychiatry. Though non-clinician-led SMAs have been applied in myriad ways in primary care settings, such as group-based acupuncture clinics, group psychotherapy for post-traumatic stress disorder and group interventions for disabled adults, we excluded them to evaluate SMAs as a variation of clinician-led primary care.

To the best of our knowledge, our current review updates the evidence base to date and provides a necessary segue to patient-oriented outcomes. In the spirit of the Triple Aim, SMAs uniquely enhance patient-centered experience, thus we limited our review to settings that provide individual primary care consultation alongside the group visit. Individual consultation provides a reserved space for private concerns. This is an important distinction as privacy concerns have been a prominent drawback of the model identified by prior research [13, 15, 34]. We prioritized this element, recognizing the trust it fosters in the patient-clinician relationship.

### Summary of findings

In sum, designing, promoting, and running SMAs from tested and proven formats proves to be vital for implementation. Model and content fidelity demonstrate significant outcome improvement, most notably in the prenatal care and birth outcomes through the CenteringPregnancy® group process. Standardized training also improves facilitation of group care. Therefore, clinicians learning to facilitate group care are encouraged to receive training in facilitative leadership with emphasis on the role that a participatory atmosphere has in improving outcomes [50].

Several models describe a physical design component to enhance the effect on patient experience or group process [3, 42, 51]. Some studies use displayed patient biophysical data for comparison and a visual aid for decision-making. Patient seating design has also been identified as a driver, both circular and U-shaped formats. Krzywokwski-Mohn stipulates that SMAs occur with participants seated around a circular conference table, with no one at the “head of the table,” balancing power and significantly influencing SMA participant outcomes [42].

Additionally, the emergence of the patient-centered medical home motivates improvement in patient education, experience of care, and measurable outcomes without increasing clinical workload [3]. The interprofessional team plays a prominent role in SMAs across the literature, including nurses, nutritionists, NPs, pharmacists, physical therapists, PAs, primary healthcare coordinators and nurse midwives [7, 8, 14, 34, 52]. Despite these reallocation of tasks, roles, and resources, SMAs demonstrate efficacy and feasibility across a wide range of healthcare systems [39, 53].

Despite SMAs objectively providing patients more time with their clinicians, the degree to which this affects satisfaction is unknown and patient characteristics and outside influences can affect satisfaction outcomes [7, 13, 49, 54]. Furthermore, evaluating and effectively responding to the social determinants of health requires improved identification of patient needs and outcomes assessment [55]. Nonetheless, our evaluation includes consideration of patient experience fundamental for evaluating health-related quality of life, including disease-related health locus of control, health behaviors, self-efficacy, and other measures of patient perspective of care and quality of life.

Lastly, studies emphasizing biophysical outcomes report statistically significant improvement in at least one biophysical metric, yet are too heterogeneous to compare across studies. Nonetheless, results are consistent with other systematic reviews by Booth et al., Edelman et al., and Jones et al. [17, 18, 49].

### Limitations of review

Our inclusion criteria and focus on the primary care context limited the number of articles that we evaluated in this review, which may impact the generalizability of our conclusions. Previous systematic reviews looked at a broader number of articles, though their approach also introduced more heterogeneity [17, 18, 49]. Single center studies, representing the majority for our included articles on diabetes patients, may also limit generalizability. We also note that half of our included articles for the SMA / GV format were authored by the same researcher [36–40]. Other previous reviews have mentioned the impossibility of blinding the participant and clinician / care team. Given that trials of SMA interventions cannot be designed in a traditional double-blinded manner, our quality assessment scores for quantitative studies could only receive a maximum of seven out of a total of eight points on the modified Jadad score. However, a few studies described minimizing performance bias by having the same clinician and care team manage the same intervention and control subjects and by measuring outcomes blindly for the treatment group. Furthermore, there may be sampling bias in nonrandomized controlled trials as well as focus groups and interviews due to the possibility that patients who are high frequency attenders may self-select to be included in the intervention group; likewise, subjects who have negative experiences with SMAs may decline to be interviewed or refuse to be randomized into the intervention group. Moreover, information bias may have appeared due to variation in attendance and/or completion of visits within our sample.

Critiques exist concerning the evaluation of patient experience through patient satisfaction measures. Aside from a lack of agreement on a converging definition of “satisfaction,” there are methodological challenges in reliably and precisely measuring and interpreting perceptions of the healthcare environment (survey content, mode and timing of survey administration, bias, confounding, need for post-hoc adjustment, and subjective nature of interpersonal experiences, including patient-clinician communication as a unique dimension of quality). Despite these challenges, patient experience has a meaningful role in quality improvement discussions and determination of perceived quality and sense of community [56].

### Implications for practice, policy, and future research

Improved resilience and coping skills, in concert with patient agency and activation, are valuable outcomes of the spectrum of SMAs [34]. The primary care environment is an optimum setting to build the necessary trust, health literacy, and awareness of health beliefs required for successful intersection with the broader healthcare system [35, 38]. Honoring adult learning strategies often requires nonclinical skill sets for interdisciplinary care clinicians [38]; yet, few studies focused on interprofessional practice despite widespread presence across differing SMA models. SMAs emphasize patient empowerment through peer accountability, socialization, and appreciation of local cultural context as well as patients’ familiarity and comfort with the setting [40, 43, 53]. Engaging group members in the design of these SMAs can maximize responsiveness to cultural context and acceptability of the model [43]. GPNC / CP have demonstrated efficacy in increasing health-related knowledge, social support, personal locus of control, emotional care, and self-care [52, 57].

In general, to improve quality and validity of reporting patient experience as well as improved reporting of population health outcomes, we recommend longer duration of follow up in each study setting. We also recommend specific evaluation of team-based care, including perspectives of administrators and supporting clinical staff. As provision of healthcare is a service, measures of quality should include assessment of the extent to which patients and care teams reach a common understanding of treatment course and health outcomes [2]. This intersection of shared well-being with health improvement warrants further evaluation to optimize healthcare delivery models, such as SMAs, to achieve the quadruple aim.

## Conclusions

Shared medical appointments are increasingly employed in primary care settings. This mixed-methods systematic review concludes that accepting and implementing this nontraditional approach by both patients and clinicians can yield measurable improvements in patient trust, patient perception of quality of care and quality of life, and relevant biophysical measurements of clinical parameters. Compared to usual care, SMAs have a greater ability to engage and empower patients as active participants in their own healthcare while improving patient access and healthcare efficiency. The cumulative benefits of SMAs are most notable when implemented within a conducive environment such as a PCMH.

No singular model of SMA best serves all settings. Similarly, there does not appear to be a priority set of outcome measures nor consistent means for their evaluation from our review. Our analysis indicates that both quantitative and qualitative methods are equally valid for evaluating patient experience. Further refinement of this healthcare delivery model will benefit from standardizing measures of patient satisfaction and clinical outcomes.

Not surprisingly, critiques and cost-benefit concerns remain. Demonstration of global payment models resulting in improved population health outcomes alongside economies of scale may be essential for wider acceptance of SMAs. We recommend further evaluation of the enablers and barriers to advance SMA integration in primary care practice settings. We also recommend more thorough and longitudinal evaluations to better describe the consumer-minded approach for care delivery design and responsiveness to the voice of the customer to achieve the most efficient models possible.

## Additional files

Additional file 1: Database search strategies (DOCX 27 kb)

Additional file 2: Description of data: Reported significant findings related to patient experience and satisfaction, as reported in included articles (DOCX 31 kb)

Additional file 3: Description of data: Barriers to implementation from available studies (no. of articles = 8) (DOCX 28 kb)

Additional file 4: Description of data: SMA patient-centered variables vs. attendance and outcomes (no. of articles = 26) (DOCX 40 kb)

Additional file 5: Inter-rater reliability of included articles using two-way mixed measures intraclass correlation (ICC) value for average agreement presented. (DOCX 29 kb)

## Data Availability

Table 1 provides a list of the 26 included papers and Additional file 1 shows the database search strategy.

## Abbreviations

CHCC: Cooperative health care clinic
CP: CenteringPregnancy®
DQoL: Diabetes-Related Quality of Life
GPNC: Group prenatal care
GV: Group visit
PCMH: Patient centered medical home
SMA: Shared medical appointment
