# Supplementary material for: Shared medical appointments and patient-centered experience: a mixed-methods systematic review
Source: BMC Fam Pract. 2019 Jul 8;20:97. doi: 10.1186/s12875-019-0972-1 (PMC6615093; doi:10.1186/s12875-019-0972-1)
Supplement: Supplementary file 1 — Database search strategies (DOCX 27 kb) [file 12875_2019_972_MOESM1_ESM.docx]

**Additional file 1**. Database search strategies

Database: PubMed
Search Date: 19 November 2015

#1 cluster clinic*[tiab] OR cluster visit*[tiab] OR cooperative health care clinic*[tiab] OR group appointment*[tiab] OR group care[tiab] OR group clinic[tiab] OR group clinics[tiab] OR group medical appointment*[tiab] OR group medical care[tiab] OR group medical clinic*[tiab] OR group medical visit*[tiab] OR group office visit*[tiab] OR group visit*[tiab] OR shared appointment*[tiab] OR shared medical appointment*[tiab] OR shared medical visit*[tiab]

#2 “Appointments and Schedules”[majr] AND (group[ti] OR shared[ti] OR cluster[ti])

#3 attitude[mh] OR attitude*[tiab] OR patient acceptance of health care[mh] OR accepta*[tiab] OR patient satisfaction[mh] OR satisf*[tiab] OR patients/psychology[mh] OR perception[mh] OR perception*[tiab] OR personal satisfaction[mh] OR health services accessibility[mh] OR accessib*[tiab] OR appreciat*[tiab] OR motivation[mh] OR motivat*[tiab] OR patient participation[mh] OR patient participat*[tiab] OR patient activation*[tiab] OR patient experience*[tiab] OR patient perspective*[tiab] OR patient opinion*[tiab] OR suitab*[tiab]

#4 (#1 OR #2) AND #3

#5 #4 AND (“1997/01/01”[PDAT] : “3000/12/31”[PDAT]) NOT (“child”[MeSH Terms] NOT “adult”[MeSH Terms])

Database: Cochrane Library (Wiley)
Search Date: 12 November 2015

ID Search

#1 cluster next (clinic? or visit?)

#2 “cooperative health care” near/2 clinic?

#3 group next (appointment? or care or clinic? or visit?)

#4 shared near/2 (appointment? or visit?)

#5 [mh “appointments and schedules”] and (shared or group or cluster):ti

#6 #1 or #2 or #3 or #4 or #5

#7 MeSH descriptor: [Attitude] explode all trees

#8 MeSH descriptor: [Health Services Accessibility] explode all trees

#9 MeSH descriptor: [Motivation] explode all trees

#10 MeSH descriptor: [Patient Participation] explode all trees

#11 MeSH descriptor: [Patient Satisfaction] explode all trees

#12 MeSH descriptor: [Personal Satisfaction] explode all trees

#13 MeSH descriptor: [Perception] explode all trees

#14 MeSH descriptor: [Patient Acceptance of Health Care] explode all trees

#15 MeSH descriptor: [Patients] explode all trees and with qualifier(s): [Psychology - PX]

#16 #7 or #8 or #9 or #10 or #11 or #12 or #13 or #14 or #15

#17 accepta* or accessib* or appreciat* or attitude* or motivat* or suitab* or satisf* or perception?

#18 (patient next activation?) or (patient next experience?) or (patient next perspective?) or (patient next opinion?) or (patient next participat*)

#19 #16 or #17 or #18

#20 #6 and #19 Publication Year from 1997 to 2015

Database: PsycINFO (EBSCO)
Search Date: 11 December 2015

S1 ( cluster W1 (clinic OR visit) ) OR cooperative health care clinic OR ( group W1 (appointment OR care OR clinic OR visit) ) OR ( shared W1 (appointment OR visit) )

S2 DE “Client Attitudes” OR DE “Client Satisfaction” OR DE “Client Participation” OR DE “Gratitude” OR DE “Motivation” OR DE “Satisfaction” OR DE “Perception”

S3 TI accepta* OR AB accepta* OR TI accessib* OR AB accessib* OR TI appreciat* OR AB appreciat OR TI motivat* OR AB motivat* OR TI patient activation* OR AB patient activation* OR TI patient experience* OR AB patient experience* OR TI patient opinion* OR AB patient opinion* TI patient perspective* OR AB patient perspective* OR TI suitab* OR AB suitab* OR TI satisf* OR AB satisf* OR TI perception* OR AB perception*

S4 S2 OR S3

S5 S1 AND S4 (Limiters - Publication Year: 1997-2015; Peer Reviewed)

Database: CINAHL (EBSCO)
Search Date: 11 December 2015

S1 ( cluster W1 (clinic OR visit) ) OR cooperative health care clinic OR ( group W1 (appointment OR care OR clinic OR visit) ) OR ( shared W1 (appointment OR visit) ) OR ( (MH “Appointments and Schedules”) AND (TI group OR TI shared OR TI cluster) )

S2 MH “Attitude+” OR MH “Consumer Participation” OR MH “Motivation+” OR MH “Patient Satisfaction” OR MH “Health Services Accessibility+” OR MH “Personal Satisfaction” OR MH “Perception+” OR MH “Patients+/PF”

S3 TI accepta* OR AB accepta* OR TI accessib* OR AB accessib* OR TI appreciat* OR AB appreciat OR TI attitude* OR AB attitude* OR TI motivat* OR AB motivat* OR TI patient activation* OR AB patient activation* OR TI patient experience* OR AB patient experience* OR TI patient opinion* OR AB patient opinion* OR TI patient participat* OR AB patient participat* OR TI patient perspective* OR AB patient perspective* OR TI suitab* OR AB suitab* OR TI satisfaction* OR AB satisfaction* OR TI perception* OR AB perception*

S4 S2 OR S3

S5 S1 AND S4

S6 S5 NOT (MH child+ NOT MH adult+) (Limiters - Published Date: 19970101-; Peer Reviewed)

Database: Web of Science
Search Date: 28 January 2016

Timespan=1997-2016. Indexes=SCI-EXPANDED, SSCI, A&HCI, ESCI.*

#1 TS=(cluster NEAR/1 (clinic? or visit?))

#2 TS=“cooperative health care” NEAR/0 clinic?

#3 TS=(group NEAR/1 (appointment? or clinic? or visit?))

#4 TS=(shared NEAR/1 (appointment? or visit?))

#5 TS=appointments

#6 TI=(group OR shared OR cluster)

#7 #5 AND #6

#8 #1 OR #2 OR #3 OR #4 OR #7

#9 TS=(attitude* OR accepta* OR satisf* OR perception* OR accessib* OR appreciat* OR motivat* OR “patient participat*” OR “patient activation*” OR “patient experience*” OR “patient perspective*” OR “patient opinion*” OR suitab*)

#10 #8 AND #9

Database: ClinicalTrials.gov
Search Date: 5 August 2016

#1 “cluster clinic” OR “cluster visit” OR “cooperative health care clinic” OR “group appointment” OR “group care” OR “group clinic” OR “group medical appointment” OR “group medical care” OR “group medical clinic” OR “group medical visit”

#2 “group office visit” OR “group visit” OR “shared appointment” OR “shared medical appointment” OR “shared medical visit”

(Manually combined results of #1 and #2)

Database: SSRN
Search Date: 4 December 2015

“cluster clinic”

“cluster clinic”

“cluster visit”

“cluster visits”

“cooperative health care clinic”

“cooperative health care clinics”

“group appointment”

“group appointments”

“group care”

“group clinic”

“group clinics”

“group medical appointment”

“group medical appointments”

“group medical care”

“group medical clinic”

“group medical clinics”

“group medical visit”

“group medical visits”

“group office visit”

“group office visits”

“group visit”

“group visits”

“shared appointment”

“shared appointments”

“shared medical appointment”

“shared medical appointments”

“shared medical visit”

“shared medical visits”

Database does not permit Boolean operator OR. Each term searched individually.
