# Supplementary material for: Shared medical appointments and patient-centered experience: a mixed-methods systematic review
Source: BMC Fam Pract. 2019 Jul 8;20:97. doi: 10.1186/s12875-019-0972-1 (PMC6615093; doi:10.1186/s12875-019-0972-1)
Supplement: Supplementary file 2 — Description of data: Reported significant findings related to patient experience and satisfaction, as reported in included articles (DOCX 31 kb) [file 12875_2019_972_MOESM2_ESM.docx]

**Additional file 2**. Reported significant findings related to patient experience and satisfaction, as reported in included articles

| **Article** | **Collection Style** | **Reported Significant Findings re: Patient Perceptions of Care** |
| --- | --- | --- |
| Clancy, 2007 | Primary Care Assessment Tool (PCAT) / Trust in Provider Outcomes | SMA patients had improvement in PCAT domains of cultural competency and community orientation; also had significantly improved perception of continuity of care. SMA patients were more likely to report the health professional as a powerful locus of control. (p312) |
| Clancy, 2003 | Primary Care Assessment Tool (PCAT) / Trust in Provider Outcomes | SMA patients were more likely to say their questions were answered in ways they understood, that their PCP gave advice about healthy foods, and that they would recommend their PCP. Also more likely to say their PCP was trustworthy and that they tried to follow PCP’s advice, that they trust their provider’s judgments about medical care, and that they would trust their provider to say if there was a mistake made. (p300) |
| Andersson, 2012 | 1:1 Phone or in-person interviews; Focus Group Style Interviews | Themes: The care – combining individual physical needs w/ preparation for parenthood (p504-5); the group – a composed recipient of care (p505-6); the midwife – a controlling professional (p506-7) |
| Raballo, 2012 | 1:1 Phone or in-person interviews | SMA patients had more positive attitudes and did not show development of negative empowerment; more external LOC with usual care and internal LOC w/ group care. Usual care visits were described with words including “Tension,” “Anxiety,” and “Fear” while SMA patients used words like “Knowledge,” “Educational” and “Friendship.” (p245) |
| McDonald, 2014 | Focus Group Style Interviews | Themes: Connecting and networking (p4), Education and preparation (p5), Time and efficiency (p5), Making connections (p5), Learning from the group (p6), Normalizing the pregnancy experience (p6), improved relationships w/ midwives (p6), feeling prepared for L+D (p6-7). There were also reports of increased time with care providers and more information, though the providers were not always continuous (p10). Concerns: initially there was concern of not having enough individual time w/ provider (p7) and constructive feedback was offered with respect to program content (p7-8), physical environment (p8), access to one’s own midwifery team (p8), participation of midwifery students (p8-9) and scheduling difficulties (p9). |
| Beck, 1997 | 1:1 Phone or in-person interviews | SMAs had higher proportion of patients rating care as “excellent.” They were also and were more likely to say they could obtain appointments “as soon as I would like,” and that that “all healthcare needs were met.” (p548) |
| Junling, 2015 | Self efficacy / participation / satisfaction questionnaires | SMA participants enjoyed greater increases in Self-Efficacy in managing symptoms, Self-Efficacy in managing disease in general, and Self-Efficacy in taking part in Physical activities than their control group counterparts (p356). SMA patients also experienced more positive changes on measurements of depression, health distress, Patient-physician communication, social support, and coping skills (p357). |
| Scott, 2004 | Self efficacy / participation / satisfaction questionnaires | SMA patients had higher scores on patient satisfaction scales of: satisfaction w/ PCP, physician’s unhurriedness, time spent, and overall quality of care (p1467). These patients also reported a higher self-efficacy in communication with physicians, as well as higher overall quality of life (p1468). |
| Trento, 2001 | Modified DQOL | SMA patients showed improved scores on the DQOL/Mod, while there were no changes in score among the control subjects (p997-8). |
| Trento, 2002 | Modified DQOL | SMA patients showed improved scores on the DQOL/Mod, while scores worsened amongst control patients.  Differences remained significant after adjustment for age, duration of disease, education (p1235) |
| Trento, 2004 | Modified DQOL | SMA patients showed an improvement in QOL after two years; after year two this variable had worsened among control subjects (p672). |
| Trento, 2005 | Modified DQOL | SMA patients had improved QOL while QOL worsened amongst control patients (p297). |
| Trento, 2010 | Modified DQOL | QOL improved in SMA subjects and worsened in control subjects (p746-7). At study end, QOL was better in SMA subjects (p747). |
| Naik, 2011 | Modified DQOL | Diabetes self efficacy measures improved immediately after intervention compared to baseline in both intervention groups; efficacy measures at 3 months were significantly higher in the SMA intervention compared w/ those in the education intervention. Note: self-efficacy scores returned to baseline levels at 1 year; w/ modest (nonsignificant) between-group distances (p6). |
| Kennedy, 2011 | Self efficacy / participation / satisfaction questionnaires | Women enrolled in GPC were significantly more likely to be satisfied with their care and felt they were more able to participate than those in IPC (p1174). |
| Tandon, 2013 | Self efficacy / participation / satisfaction questionnaires | Women in CP groups were more satisfied with their prenatal care, and more actively participated in their prenatal care, than women receiving traditional care.  CP patients were more satisfied with time spent talking w/ provider, ability to talk to their prenatal provider in language they were most comfortable, and acceptability of prenatal care visit waiting time (p1059).  Note: 90% of CP patients received adequate or adequate plus prenatal care, compared w/ 63% of traditional care participants (p1059). |
| Jafari, 2010 | Self efficacy / participation / satisfaction questionnaires | SMAs: more satisfied with information received during prenatal care, coordination of care, relationship with provider, and quality of care; felt better informed about labor and delivery, self-care during pregnancy, family planning, care of newborn, nutrition during pregnancy, problems during pregnancy, prenatal medical procedures and tests, and breast feeding. They were also more likely to say their provider listened to their problems and answered all their questions, and that their care was provided in a supportive and confident way. Also more satisfied w/ the time their providers spent with them during visits, the arrangements for making appointments, and waiting time, as well as the completeness of their physical exam, competence of their provider, and overall quality of prenatal care (p55). |
| Capello, 2008 | 1:1 Phone or in-person interviews | Themes: Ability to talk to physician; learning about dietary guidelines and exercise; encouraging patients to explore what it takes to make changes; hearing other members talk about shared problems; receiving lots of information; information about various medications; meeting new people; discussing ways to handle stress; direct access to healthcare providers. (p126).  Areas for improvement: more handouts; more direction; more continuance; more interactions between members (p127). |
| Wong, 2015 | 1:1 Phone or in-person interviews | Themes: GMVs foster access to needed health services, expanded opportunities for collaboration and team-based care, and improved patient and provider experiences. (p33-6). SMA patients reported increased healthcare management skills within their own personal contexts, and also described being “motivated by knowing they were ‘not alone,’” and felt that SMAs neutralized the inherent power balance between patient and provider (p36). |
| Herrman, 2012 | Focus Group Style Interviews | Major ideas discussed: Program (p21); People (p21); Information (p22); Support (p23); Health Behaviors (p3).  Themes/Discussion: It’s about respect (p23-4); Knowledge is power (p24); I’m a better mother (p24); Supporting each other (p24). |
| McNeil, 2012 | 1:1 Phone or in-person interviews | Themes: Getting more in one place at one time (p4-5); Feeling supported (p5); Learning and gaining meaningful information (p5); Not feeling alone in the experience (p5); Connecting (p6); Actively participating and taking ownership of care (p6). |
| Novick, 2011 | 1:1 Phone or in-person interviews | Themes: Investment – “Sitting there taking time out” (p8-9); Collaborative Venture – “Everybody talked to everybody” (p9-10); Social Gathering – “You’re talking and you’re laughing” (p10-11); Relationships with boundaries – “We had a little moment” (p11-4); Learning in the group – “Learning so you understand” (p14-6); Changing self – “It was not just me, and the world’s not over” (p16-8). |
| Krzywkowski-Mohn, 2008 | 1:1 Phone or in-person interviews | Themes: Supportive caring venue (p150-2); Opportunity to speak regarding experiences (p152-3); Humor (p153); Education and knowledge needed to understand diabetes (p153-5); Preventing isolation (p155-6); Agent Orange and Vietnam Veterans (p156-7); Mental health issues (p157-8); Sexual dysfunction (p158-9); Administration supportive (p159-61) |
| Heberlein, 2016 | 1:1 Phone or in-person interviews | Women attending SMAs described that the “education and preparation” function of SMAs was enhanced through the development of relationships with other women in their groups (p227). SMA patients also described that the extra time in sessions helped them to develop solid relationships with their providers (p228). Nulliparous SMA patients reported “reduced stress and improved confidence” regarding the postpartum period due to education they had received at their visits, as well as developing supportive relationships with their fellow group-goers (p 231). |
| Andersson, 2013 | Self efficacy / participation / satisfaction questionnaires | There was no difference between models of care regarding overall satisfaction; the majority in both groups reported positive opinion about overall assessment of antenatal care (p117). Participants in SMAs were more satisfied when the care they received supported contact with other parents (p118). |
| Kennedy, 2009 | 1:1 Phone or in-person interviews | Themes: “I wasn’t alone“ – the experience with group PNC (p179-80); “I liked it but…” – recommendations to improve group PNC (p180); “They really needed to listen” – general concerns across the sample about their childbearing care (p180-1). Overall high enthusiasm for group care from most but not all participants (p 178). |
