# Supplementary material for: Shared medical appointments and patient-centered experience: a mixed-methods systematic review
Source: BMC Fam Pract. 2019 Jul 8;20:97. doi: 10.1186/s12875-019-0972-1 (PMC6615093; doi:10.1186/s12875-019-0972-1)
Supplement: Supplementary file 3 — Description of data: Barriers to implementation from available studies (no. of articles = 8) (DOCX 28 kb) [file 12875_2019_972_MOESM3_ESM.docx]

**Additional file**. Barriers to implementation from available studies (no. of articles = 8)

| **First author, year** | **Model** | **Sample size** | **Group visit attendance** | **Barriers / limitations identified** |
| --- | --- | --- | --- | --- |
| Beck, 1997 | CHCC | 160 group (n=160); 161 control (n=161) | 55% total attendance;  (6.62/12) mean visits / 1 yr | - Patient lack of interest, attrition from group. |
| Scott, 2004 | CHCC | 146 CHCC (n=146); 149 control (n=149) | 40.8% total attendance;  (10.6/24) mean visits / 2 yr;  25.5% attended 2 or fewer | - Frail patients show poor attendance. - Economic advantages depend on group size. - Required monitoring to maintain patient interaction. - Some patients are uncomfortable with group interaction. - Some physicians are uncomfortable leading group discussions. - Benefits are invisible to clinic staff making ongoing support difficult. |
| Junling, 2015 | CHCC | 692 group, 92 LTFU (n=600); 654 control, 50 LTFU (n=604) | 90% total attendance;  (5.4/6) mean intensive visits; 72.3% attended all 6;  (4.8/6) mean continuous visits; 66.2% attended all 6 | - Many physicians feel the traditional individual appt model, for which they are trained, is the best form of care. - Patient and provider attitudes toward group model must improve for acceptance. - Content of the group visit must meet patient needs. - Training for group visit is necessary before implementation. - Key factor is support of community committee (Chinese community sys). |
| Naik, 2011 | SMA | 45 EPIC group, 1 LTFU (n=44) 42 traditional, 1 LTFU (n=41) | Unknown total attendance;  96% attended some or all (4 sessions/3 months);  51% (23/45) attended all sessions, 44% (20/45) attended some, 0.04% (2/45) did not attend any | - Duration of the EPIC intervention sessions exceeded the length of instruction in the traditional groups. - Significant time and effort required by patients and clinicians to attend multiple sessions over three months. - Additional training required of study clinicians. |
| Wong, 2014 | SMA | 34 providers and 29 patients | Avg 4 SMAs / 1 yr (range 1 - 15). Offered weekly to quarterly | - Structural challenges in delivering SMAs. |
| Kennedy, 2009 | CP | 322 pregnant military women enrolled in study; n=234 completed the final 3-month post-partum interview | 72.6% (234 / 322) women enrolled completed the final 3-month postpartum interview | - Some women identified a desire for more individual time with the provider and more privacy. |
| McDonald, 2014 | CP | 9 women and 5 midwives. Purposive criterion sampling for low-risk women in GPC at a midwifery clinic. | Not reported | - Challenges of scheduling and system level issues though facilitated by flexibility and commitment to the model. |
| Novick, 2011 | CP | 45 interviews of n=21 pregnant women who consented to indiv interviews; n=18 subjects of participant observation (8 pregnant women declined interview, 6 guests [3 men, 3 women], 2 CNM group leaders, and 2 medical assistants from 4 'Centering' groups) | 43% (4.3/ 10 mean visits);  54% (5.4 / 10 mean visits for principle participants) | - Important boundaries on relationships between participants, and some women wished for greater privacy during physical examinations. |

LTFU = lost to follow-up
