# Supplementary material for: Shared medical appointments and patient-centered experience: a mixed-methods systematic review
Source: BMC Fam Pract. 2019 Jul 8;20:97. doi: 10.1186/s12875-019-0972-1 (PMC6615093; doi:10.1186/s12875-019-0972-1)
Supplement: Supplementary file 4 — Description of data: SMA patient-centered variables vs. attendance and outcomes (no. of articles = 26) (DOCX 40 kb) [file 12875_2019_972_MOESM4_ESM.docx]

**Additional file 4**. SMA patient-centered variables vs. attendance and outcomes (no. of articles = 26)

| **First author, year** | **Duration of group visits x no. of appts / yrs of follow up = Dosage (mins/yr)** | **Pt language, culture, SE status, reimbursement** | **No. of patients in each group (mean or range)** | **SMA attendance %** | **Key findings** |
| --- | --- | --- | --- | --- | --- |
| ***CHCC (5 articles)*** | | | | | |
| Beck, 1997 | 120 mins * 12 visits / 1 yr =  1440 mins/yr | Medicare patients; HMO | 8 (mean) | 55% attendance of total CHCC's scheduled; 6.62 avg group meetings | - Reduced repeat hospital admissions, emergency care use and cost of care - More effective delivery of certain preventive services - Increased patient and physician satisfaction |
| Clancy, 2003 | 120-mins * 6 monthly visits / 1 yr =  720 mins/yr | Uninsured, under-insured adults with T2DM; 62% African American, 36% live outside area served by public transport; 65% female. | 19 to 20 | Overall= 69%; Group 1= 59%; Group 2= 65%; Group 3= 82%; | - Patients who received group care showed improved sense of trust in their physician compared to usual care. - Additional tendency of group participants to report greater coordination, community orientation, and more culturally competent care. - Attendance indicated good acceptance. |
| Clancy, 2007 | 120 mins * 12 visits / 1 yr = 1440 mins/yr | Uninsured, under-insured | 14 - 17 | 70.46% attendance rate overall; 27 withdrew, 3 died | - Group scored higher in PCAT domains of ongoing care, community orientation, and cultural competence compared to control. - Group patients scored higher in Powerful-Other Health Professional subscale of DLC. |
| Junling, 2015 | 120 mins * 9 visits = 1080 mins/yr  [120 mins * 2  monthly (mos 1-3), 120 mins monthly (mos 4-6)] | Chinese nat’l health system | 18 - 20 (encouraged) | 90% overall in group visit. Patients attended avg of 5.4 of 6 intensive session visits (72.3% attended all 6) and 80% overall in control. 4.8 of 6 continuous usual session visits (66.2% attended all 6) | - - Significant decreases in systolic and diastolic blood pressure equal or greater than that seen in controls. - BMI unchanged in intervention or control groups. - Significant increases in physical activity, medication and dietary compliance in group compared to controls. - Significant increases in health and energy, self-efficacy in managing symptoms (SEMS), disease in general (SEMDG) and physical activities (SEPA) in group compared to control |
| Scott, 2004 | 150 mins * 24 visits / 2 yrs = 1800 mins/yr | Medicare; chronically-ill; HMO | 6 to 12 | 40.8% total attendance at group visits (Mean 10.6 group visits); 25.5% attended 2 or fewer group visits | - Mean of 8 patients attended each of 10 CHCC meetings over 24 months. - Mildly reduced costs. - Significantly improved patient satisfaction, self-efficacy for communication, and QOL. |
| ***SMA / GV (10 articles)*** | | | | | |
| Capello, 2008 | 90 min s* 4 weekly visits = 360 mins/yr | Adult, male, military veterans | unclear, varies | 72 completed initial questionnaire; 58 (81%) continuation through completion; other absenteeism was not reported but writing indicates some may have completed only 80% of meetings but were still included in the analysis through mean data substitution | - Group visits showed significant effects on systolic and diastolic blood pressure. - Group visits showed significant changes in reported health-behaviors, outlook, and coping mechanisms. |
| Krzywkowski-Mohn, 2008 | 120 mins * at least 3 visits = 360+ mins/yr | Male veterans in the Cincinnati VA medical system; 16 white, 16 black, 1 Native American | 8 to 12 | 64% completion;  21 of 33 participants attended at least 3 meetings | - Significantly improved control of diabetes and cholesterol; 28% increase in retinal examination. - Similar high rates of diabetic foot exams. - Supporting and caring venue, - Opportunity to share experience, - Humor/education/knowledge of disease process, - Prevent isolation, mental health issues, sexual dysfunction, - administrative support. |
| Naik, 2011 | 180 mins * 4 group sessions every 3 wks over 3 mos =  720 mins/yr | Veterans | 5 to 7 | 51% overall attendance in EPIC - Of 45 patients, 23 attended all sessions, 20 (44%) attended some, 2 (4%) did not attend any | - EPIC intervention showed significantly greater improvements in HbA1c levels immediately following the active intervention and these differences persisted at 1 year follow-up - Diabetes self-efficacy measures at 3 months were significantly higher in the EPIC vs. education intervention, and scores returned to baseline levels at the 1-year data collection. |
| Raballo, 2012 | unclear | Italian diabetics attending Turin University diabetes clinic | 6 to 10 | Avg length of participation in group care for T1DM pts was 6.6 yrs [4.1, 9.1], and 9.5 yrs [5.3, 11.7] for T2DM pts. | - Mostly positive attitudes in group care patients. Negative empowerment not seen in group care vs. control. - More internal locus of control in group care vs external in control. - Group care articulated larger range of care concepts. - Positive attitudes associated with group care regardless of diabetes type. |
| Trento, 2001 | 60 mins * 8 visits / 2 yrs = 240 mins/yr | Italian nat’l health system | 9 to 10 | 99% avg 7.9/8 (range 7-8) group visits during 2 years vs 103% avg 8.2 individual visits (range 5-11) | - Group visit clinicians must provide information appropriate to adult learning styles. - Managing T2DM patients through group visits may be a feasible and more efficient alternative. |
| Trento, 2002 | 60 mins * 4 visits / 1 yr (years 1 - 2); Repeated curriculum 60 mins * 7 visits / 2 yrs (years 3 - 4) = 240 mins/yr | Italian Type 2 diabetics, Italian Nat'l Health System | 8.4 patients avg (groups of 9-10 pts) | 80% = 45 of 56 patients in group care completed the study (3 died, 8 moved to different clinics; 2 patients left groups in year 1 for personal reasons but rejoined in year 3) | - Group intervention showed significant decrease in HbA1C, improved QOL, Knowledge of Diabetes, and health behaviors, reduced dosage of anti-hyperglycemic agents and rates of retinopathy. - Similar reductions in BP and CV risk in group vs control patients. - Group care required slightly more time and money than individual care, resulting in $2.12 additional spending per point gained on QOL scale. |
| Trento, 2004 | 60 mins * 4 visits / 1 yr (7 sessions spread over 2 years and repeated) =  240 mins/yr | Adult, Italian health system, diabetes patients | 8 to 12 | 75% completion of intervention = 42/56 (of 56 randomized, 14 were lost to follow-up.); 75% control = 42/56 (of 56 randomized, 14 were lost to follow-up.) | - Group visit education procedures and settings tailored to adults with T2DM allow enhanced acquisition of specific knowledge and conscious behaviors compared to traditional optimized care (associated with progressive deterioration of knowledge, problem-solving, and quality of life.) |
| Trento, 2005 | 60 mins * 15 visits / 3 yrs = 300 mins/yr | Italian nat’l health system | 6 to 7 | 95.7% attendance overall in group care vs. 83.3% attendance overall in control | - Significant improvement in quality of life, diabetes knowledge, and health behaviors in intervention group. - Quality of life worsened while diabetes knowledge and health behaviors remained unchanged in controls. - Greater cost in intervention group but showed 19.42 Euros/patient spent over 3 years improved quality of life by 1 point. - No differences in perceived satisfaction between group and individual treatment. |
| Trento, 2010 | 60 mins * 4 visits / 1 yr (7 sessions spread over 2 yrs and repeated) =  240 mins/yr | Italian health system; diabetics, < 80 yo | 8 to 12 | (815 - 106 - 128) / 815 = 71.3% completion Two clinics did not complete the trial. No individual attendance data; Case (n=106) and Control (n=128) dropouts had similar baseline variables. | - Intervention subjects had decreased BMI, fasting glycemia, A1C, total cholesterol, triglycerides, LDL cholesterol, and BP from baseline after year 4. - HDL cholesterol increased and creatinine did not change. - Health behaviors, QOL, and knowledge improved significantly in case subjects. - In controls, Health behaviors did not change and QOL decreased. |
| Wong, 2015 | 60 - 90 mins * avg 4 visits / 1yr =  240 to 360 mins/yr | Avg 62 yo, mostly female and married, majority of pts (~38% <$20,000 income) | 6 to 10 | Avg 4 visits / 1 yr (range 1 - 15).  Offered weekly to quarterly | - Foster access to needed health services; - Expand opportunities for collaboration and team-based care; - Improve patient and provider experiences. - Structural challenges in delivering group visits. |
| ***GPNC / CP (11 articles)*** | | | | | |
| Andersson, 2012 | unclear | Sweden Nat’l Health System | unclear | unclear | - Most parents felt secure in groups and said that their pregnancy symptoms were normalized with peer support. - Physical needs were perceived fulfilled and needed information was shared, although parents requested more information about the period immediately after birth. - Parents made suggestions for improving the group model of care (e.g. midwives should encourage more communication in the group setting and be more observant of men's needs in the group.) |
| Andersson, (2013) | 120 mins * (12 prenatal + 1 post-partum visits.) =  1,560 mins/yr | Sweden Nat’l Health System | unclear | Visits to midwife: Avg 9.32 in GBAC vs. 8.17 in standard care; avg visits to physician not statistically significant; 46.5% of GBAC patients lost to follow up, comparable to 50% loss to follow up in the control group. | - GBAC women reported greater satisfaction in gaining contact with other parents after group adjustment. - Group care reported greater support of initiating breastfeeding. - No significant difference in overall satisfaction between groups. |
| Heberlein, 2016 | 120 mins * 10 weekly prenatal visits / 1 yr = 1200 mins/yr | 52% black/African American; 45% white; mean age 25.5 yrs; most had a high-school educ & low-household income; 24% were married | 8 to 12 | individual care participants attended a mean of 11.1 visits; group care participants attended a mean of 9.2 sessions ( all received at least 2 other individual care appts - defined by clinical practice guidelines) | - Group care provides more and different benefits compared with individual prenatal care. Outcomes beyond health, satisfaction, and utilization measures are valuable. - Women want to maximize chance of having a healthy baby, reduce pregnancy related stress, develop confidence and knowledge for improving health; prepare for birth labor, and child care; and build supportive relationships. - Group models of prenatal care appear to be important interventions toward these goals. |
| Herrman, 2012 | 120 mins * 10 weekly prenatal visits / 1 yr = 1200 mins/yr | Pregnant women betw 18 and 35 yrs old. 23 Black non-Hispanic, 7 white non-Hispanic, and 4 Hispanic women | unclear | N/A | - Four substantive themes 1) it's about respect; 2) knowledge is power; 3) I'm a better mother; 4) supporting each other. - CP was well received by urban, low-income women during their pregnancy and may have value with select populations. |
| Jafari F, 2010 | 90 - 120 mins * 10 group visits during pregnancy =  900 to 1200 mins/yr | Iranian health system | 5 to 7 | 70.3% of women in GPC received the specified number of prenatal visits (adequacy of received services). 37.3% of the women in individual care | - Women in group visits very satisfied with prenatal care services while those in individual care were somewhat satisfied. In all measures women in group visits were more satisfied than those in the individual care. Group visit participants more likely to say provider listens to their problem and answers their questions, their care during pregnancy was provided in a supportive and confident way, and were more satisfied with friendliness and concern shown by providers. - Women in group visits more satisfied with amount of time the provider spent with them during prenatal visits, arrangements for making appointments for prenatal visits and waiting time. Women in group visits more satisfied with completeness of their physical exam, competence of provider and overall quality of prenatal care. - 37.3% of individual care women received the specified number of prenatal visits (adequacy of received services). - 70.3% of women in group visits received the specified number of prenatal visits |
| Kennedy, 2009 | 120 mins * 10 weekly prenatal visits / 1 yr = 1200 mins/yr | Pregnant women in highly mobile military communities; average age 25 years | 8 to 12 | 72.6% (234 / 322) women enrolled completed the final 3-month postpartum interview | - Military women in PNC clearly liked the CP model, but some identified a desire for more individual time with the provider and more privacy. |
| Kennedy, 2011 | 120 mins * 9 visits + 1 postpartum 120 mins reunion =  1,200 mins/yr | Military families | unclear | 53.3% in Individual care vs. 87.1% in group care overall attendance at all 9 prenatal visits | - Women in GPC more likely to receive adequate prenatal care, more likely satisfied with care, and felt they were more able to participate. - No significant difference in breastfeeding initiation or continuation. - No significant difference in PHBS or Childbirth Self Efficacy Inventory. - No significant differences in perinatal and infant health, however GPC patients were less likely to report feelings of guilt or shame. |
| McDonald, 2014 | 120 mins * 10 weekly prenatal visits / 1 yr = 1200 mins/yr | Canadian, low-risk women in group prenatal care | up to 10 | n/a | - Benefits of group learning but concerns of insufficient time with the midwife. - Suggestions for change in content, environment, partners, and access to midwives. - Challenges of scheduling and system level issues though facilitated by flexibility and commitment to the model. |
| McNeil, 2012 | 120 mins * 10 weekly prenatal visits / 1 yr = 1200 mins/yr | Postpartum women, purposively sampled. Low-risk pregnancies. | 8 to 12 | N/A | - Themes: Getting more in one place at one time, feeling supported, learning and gaining useful information, not feeling alone in the experience, connecting, actively participating and taking on ownership of care. - Core experience: getting more than they realized they needed. |
| Novick, 2011 | 120 mins * 10 weekly prenatal visits / 1 yr = 1200 mins/yr | Women who consented to individual interviews | 1 to 8 + up to 6 guests | 43% (4.3 / 10 mean visits);  54% (5.4 / 10 mean visits for principle participants) | - Six themes were identified: 1) investment, 2) collaborative venture, 3) a social gathering, 4) relationships with boundaries, 5) learning in the group, and 6) changing self. - Women were especially enthusiastic about learning in groups, about their relationships with group leaders, and about having their pregnancy-related changes and fears normalized. - Important boundaries on relationships between participants, and some women wished for greater privacy during physical examinations. |
| Tandon,  2013 | 120 mins *10 to 12 group visits = 1200 to 1440 mins/yr | Hispanic; Spanish-speaking or bilingual; public health clinics | 9 to 12 | Unclear total attendance; 90% of eligible participants completed follow-up surveys | - Significant increase in satisfaction - Increased time talking with provider - Increased prenatal care utilization - More likely to establish medical homes for mother and child - Acceptable waiting time |

Abbreviations: CHCC=cooperative health care clinic; CP=CenteringPregnancy®; GPNC=group prenatal care; GV=group visit; SMA=shared medical appointment.
