# Supplementary material for: Shared medical appointments and patient-centered experience: a mixed-methods systematic review
Source: BMC Fam Pract. 2019 Jul 8;20:97. doi: 10.1186/s12875-019-0972-1 (PMC6615093; doi:10.1186/s12875-019-0972-1)
Supplement: Supplementary file 5 — Inter-rater reliability of included articles using two-way mixed measures intraclass correlation (ICC) value for average agreement presented. (DOCX 29 kb) [file 12875_2019_972_MOESM5_ESM.docx]

**Additional file 5**. Inter-rater reliability of included articles using two-way mixed measures intraclass correlation (ICC) value for average agreement presented

| **Jadad-modified Score** | | |  | **Trustworthiness Score** | | |
| --- | --- | --- | --- | --- | --- | --- |
| ***QUANTITATIVE*** | | |  | ***QUALITATIVE*** | | |
|  | ***Overall Score*** | |  |  | ***Overall Score*** | |
| **Article** | **Rater 1** | **Rater 2** |  | **Article** | **Rater 1** | **Rater 2** |
| Beck, 1997 | 4/8 | 4/8 |  | Andersson, 2012 | 12/12 | 12/12 |
| Clancy, 2007 | 5/8 | 5/8 |  | Andersson, 2013 | 10/12 | 11/12 |
| Jafari F, 2010 | 5/8 | 5/8 |  | Capello, 2008 | 11/12 | 11/12 |
| Junling, 2015 | 5/8 | 5/8 |  | Clancy, 2003 | 11/12 | 11/12 |
| Kennedy, 2011 | 4/8 | 4/8 |  | Herrman, 2012 | 7/12 | 8/12 |
| Naik, 2011 | 6/8 | 6/8 |  | Kennedy, 2009 | 12/12 | 12/12 |
| Scott, 2004 | 5/8 | 5/8 |  | McDonald, 2014 | 12/12 | 12/12 |
| Tandon, 2013 | 4/8 | 3/8 |  | McNeil, 2012 | 11/12 | 11/12 |
| Trento, 2001 | 6/8 | 5/8 |  | Novick, 2011 | 9/12 | 9/12 |
| Trento, 2002 | 7/8 | 7/8 |  | Raballo, 2012 | 11/12 | 11/12 |
| Trento, 2004 | 5/8 | 5/8 |  | Wong, 2015 | 10/12 | 12/12 |
| Trento, 2005 | 6/8 | 6/8 |  | **ICC(2,k) inter-rater reliability value: 0.923** | | |
| Trento, 2010 | 5/8 | 5/8 |  |  |  |  |
| **ICC(2,k) inter-rater reliability value: 0.956** | | |  | ***MIXED METHODS*** | | |
|  |  |  |  |  | ***Overall Score*** | |
|  |  |  |  | **Article** | **Rater 1** | **Rater 2** |
|  |  |  |  | Heberlein, 2016 | **9/12** | **10/12** |
|  |  |  |  | Krzywkowski-Mohn,  2008 | **10/12** | **9/12** |
|  |  |  |  | **ICC(2,k) inter-rater reliability value: indeterminable**  (due to sample size of n=2 studies) | | |
